# The relationship between academic passions and critical thinking in a Chinese college student sample: a latent profile analysis

**DOI:** 10.3389/fpsyg.2025.1513286

**Published:** 2025-01-30

**Authors:** Shaojie Wang, Xizhen Fan, Hao Yu, Xing Yan, Jianmei Wang, Ying Liu, Yue Li

**Affiliations:** ^1^School of Education, Guangdong University of Education, Guangzhou, China; ^2^School of Preschool Education, Changsha Normal University, Changsha, China

**Keywords:** academic passions, critical thinking, harmonious passion, latent profile analysis, obsessive passion

## Abstract

**Background:**

Academic passions, including harmonious and obsessive passions, play a significant role in academic life by influencing students’ motivation, engagement, and overall academic success. Critical thinking is essential in education as it helps individuals assess, evaluate, and make informed decisions based on reasoning, which is crucial for academic growth and lifelong learning. Given the increasing emphasis on developing critical thinking skills in education, it is crucial to investigate how academic passions influence this cognitive process in the Chinese context.

**Methods:**

A total of 698 valid questionnaires were collected from college students from Guangdong and Hunan provinces in China. This study used latent profile analysis (LPA) to investigate the profiles of harmonious and obsessive passions. Then it tested differences in critical thinking based on the profiles of harmonious and obsessive passions.

**Results:**

Three latent profiles for two academic passions were identified: low, medium, and high. Respondents in the high harmonious and obsessive passion profiles exhibited the significantly highest critical thinking abilities.

**Conclusion:**

The results confirm the heterogeneity of harmonious and obsessive passions. Students with higher levels of academic passions demonstrate enhanced critical thinking abilities compared to their peers. This study suggests that educators should pay attention to students’ academic passions when cultivating their critical thinking skills. This study offers several implications for practice. First, colleges should provide targeted counseling and guidance based on the types of students’ passion. Second, it is necessary to balance the relationship between harmonious and obsessive passions to promote students’ mental health and academic persistence. Third, colleges should help students develop harmonious passion, thereby improving their critical thinking ability.

## 1 Introduction

### 1.1 Academic passions

Passion is a strong inclination toward a personally meaningful and highly valued activity that one loves, finds self-defining and to which substantial time and energy is invested (Vallerand and Paquette, 2024; Rahimi et al., 2023). In the dualistic model of passion first proposed by Vallerand et al. (2003), harmonious passion refers to an autonomous internalization that leads individuals to choose to engage in the activity that they like. Moreover, obsessive passion refers to a controlled internalization of an activity in one’s identity that creates an internal pressure to engage in the activity that the person likes (Vallerand et al., 2003). Specifically, in the context of education, academic passions are profound variables affecting students’ performances in school (Bélanger and Ratelle, 2021; Rahimi et al., 2023). First, academic passions improve class participation and academic performance by stimulating students’ intrinsic interest and sense of engagement (Gkorezis et al., 2021; Kaoropthai, 2024). In particular, harmonious passion enables students to find a sense of accomplishment in learning, and maintain a positive state for a long time. Second, students with academic passions use self-regulated learning strategies more effectively (Lex et al., 2022; Sverdlik et al., 2022), such as time management and goal setting. This ability not only improves students’ independence, but also enhances their ability to solve complex academic tasks (Theobald, 2021). At the same time, harmonious academic passion also helps students maintain mental health in the face of academic pressure by enhancing their resilience and adaptability to challenges (Rahimi et al., 2023; Vallerand and Paquette, 2024) and avoiding burnout or withdrawal.

Many studies have investigated academic passions using variable-centered approaches, such as regression analysis and factor analysis, which ignore sample heterogeneity. It is meaningful to further studies using person-centered approaches, such as latent profile analysis (LPA), which uncovers latent profiles or groups of individuals who share a meaningful and interpretable pattern of responses on the measures of interest (Ferguson et al., 2020). Subsequent studies have examined the profiles of the dualistic model of work passion using person-centered approaches, such as LPA. Morin et al. (in press) identified four work passion profiles among employees, naming harmonious passion dominant, obsessive passion dominant, mixed-passion average, and low passion. Li et al. (2022) compared the latent profiles of entrepreneurial passion in China and South Korea. And they found that there existed non-passionate, expansive, explorative, and mature entrepreneurs in the Chinese sample, and non-passionate, conservative, expansive, and growing entrepreneurs in the South Korean sample. Li et al. (2020) also identified three work passion profiles, which were dual passion, pro harmonious passion, and pro obsessive passion.

In a word, numerous studies have confirmed the heterogeneous nature of latent work passion (e.g., Astakhova et al., 2024; Newman et al., 2021). However, few studies have focused on it in the academic condition, i.e., the academic passion. Among them is the study by Bélanger and Ratelle (2021). They identified four profiles, that is, high harmonious and obsessive passions, moderate harmonious and obsessive passions, low harmonious and obsessive passions, and high harmonious passion and low obsessive passion in a sample of 460 university students. In the current study, we adopt a similar purpose to explore the latent profiles of academic passions with LPA for each type of the academic passions separately, in order to uncover the hidden nature of these two passions in the dualistic model of passion and their relationships with critical thinking.

### 1.2 Critical thinking

According to the organization for Economic Co-operation and Development (OECD), critical thinking is one of the key skills for the complex and globalized economies and societies of the 21st century (OECD, 2024). Based on the definition from The Foundation for Critical Thinking (2024), critical thinking is that mode of thinking – about any subject, content, or problem – in which the thinker improves the quality of his or her thinking by skillfully taking charge of the structures inherent in thinking and imposing intellectual standards upon them. Critical thinking significantly improves the efficiency and quality of students in solving academic problems by training them to analyze, evaluate and synthesize complex problems (Almulla, 2023; Shanta and Wells, 2022). The logical analysis and reflection skills involved in critical thinking can enhance students’ creativity while helping students better manage academic pressure (Atwa et al., 2022; Güner and Gökçe, 2021), thereby achieving excellent results in academic performance. Furthermore, critical thinking helps students to remain competitive in their future academic and professional careers.

### 1.3 Relationship between academic passions and critical thinking in Chinese context

As aforementioned, critical thinking is a fundamental element affecting everyone’s performance. It is related to several variables, such as individual factors (Berestova et al., 2022; Chen and Wu, 2023; Christodoulakis et al., 2023), factors related to teachers and schools (Alsaleh, 2020; Palavan, 2020), and societal and cultural factors (Aston, 2024), etc. Theoretically, there might be a relationship between academic passion and critical thinking. First, academic passion drives students to actively engage in scholarly activities and develop critical thinking skills through in-depth exploration and academic problems-solving (Chichekian and Vallerand, 2022). Second, academic passion might promote the development of critical thinking by enhancing students’ cognitive and emotional engagement, stimulating deeper thinking and logical reasoning when dealing with complex issues (Lv et al., 2022; Namaziandost et al., 2023). Third, academic passion increases students’ academic self-efficacy and positive affect, which helps them maintain curiosity and inquisitiveness in the learning process, thus enhancing their critical thinking skills (Chang et al., 2022).

There are cultural differences in critical thinking and academic passions. For example, the differences in critical thinking between Eastern and Western students are mainly reflected in cultural values and education methods. The West focuses more on individualism and logical analysis, while the East emphasizes collectivism and harmony more (Van de Vliert et al., 2025; Wang and Wu, 2023). Western education focuses on open-ended questions and exploratory learning, while Eastern education emphasizes memorization and exam orientation. This cultural background makes Western students more inclined to openly question and debate, while Eastern students pay more attention to authority and consensus. In terms of academic passions, due to the aforementioned cultural differences, students from Eastern cultures are more motivated by their recognition of family expectations and social responsibilities (Cheong, 2021; Cheung and Park, 2021) and rely more on external incentives (such as parental recognition and social status). While students from Western cultures tend to choose a learning direction that matches their own interests, which is more motivated by an internal sense of achievement (Liu et al., 2020). However, few studies have investigated the relationship between academic passions and critical thinking in the Chinese context to date. In other words, most current studies tend to explore the performances of critical thinking and academic passions in the context of Western culture.

### 1.4 The present study and hypotheses

Academic passions and critical thinking play key roles in student development. However, few studies have explored their relationship in the context of Eastern culture. Therefore, this study aims to investigate the relationship between academic passions and critical thinking in the Chinese context using latent profile analysis (LPA). In this study, two hypotheses are tested backgrounded on aforementioned:

*H1*: Distinct latent profiles of harmonious academic passion and academic obsessive passion will be identified among college students.

*H2*: Students with higher levels of harmonious and obsessive academic passions will exhibit significantly higher critical thinking abilities compared to students with lower levels of passions.

The remainder of the article is organized as follows. First, we describe the methods used, including participants, measures, and data analysis. We then present the results, which include descriptive statistics, latent profiles for harmonious and obsessive passions, and their relationship with critical thinking. Finally, we conclude the study with a discussion, suggestions for future directions, and implications for practice.

## 2 Methods

### 2.1 Participants and procedures

Data were collected from a convenient sample of 826 college students from Guangdong and Hunan provinces in China. A total of 128 cases were excluded from further analyses for careless responding, as they failed to responded to two instructed items, requiring them to answer with a particular response option. Demographic details of participants are displayed in Table 1. Among the 698 participants, 84 were male (12.03%) and 614 were female (87.97%). The ages of participants ranged from 16 to 24 years (*M* = 19.93, SD = 1.22). Additionally, 285 participants were from urban areas (40.83%), and 413 were from rural areas (59.17%).

**Table 1:** Demographic details of participants (*N* and %).

Demographic variables				
Place of residence	Age	Gender		
		Male	Female	Total
Urban areas	18 years and lower	6 (0.86%)	30 (4.30%)	36 (5.16%)
	19 years	6 (0.86%)	65 (9.31%)	71 (10.17%)
	20 years	14 (2.01%)	89 (12.75%)	103 (14.76%)
	21 years	5 (0.72%)	55 (7.88%)	60 (8.60%)
	22 years and higher	5 (0.72%)	10 (1.43%)	15 (2.15%)
Rural areas	18 years and lower	7 (1.00%)	42 (6.02%)	49 (7.02%)
	19 years	9 (1.29%)	87 (12.46%)	96 (13.75%)
	20 years	13 (1.86%)	114 (16.33%)	127 (18.19%)
	21 years	14 (2.01%)	80 (11.46%)	94 (13.47%)
	22 years and higher	5 (0.72%)	42 (6.02%)	47 (6.73%)

The university institutional review board reviewed and approved the study procedures before the study began. Before answering scales, participants received an online information sheet assuring them of the confidentiality and anonymity of their data, and they were asked to give electronic informed consent. After that, participants were required to finish the passion scale and the Chinese version of the California critical thinking disposition inventory, which are introduced as follow. Upon completing scales, participants received an electronic red packet with 5 yuan RMB.

### 2.2 Measures

#### 2.2.1 Academic passions

Academic passions were assessed using the Chinese version of the passion scale (Marsh et al., 2013; Zhao et al., 2015). This scale comprises two subscales, measuring harmonious passion and obsessive passion, respectively. Harmonious passion is a strong desire to freely engage in the activity (six items; e.g., “my studies are in harmony with the other activities in my life,” and “我的学业活动和我生活中的其他所有活动是和谐共存的” in Chinese). Obsessive passion refers to an uncontrollable urge to partake in the favorite activity (six items; e.g., “I have difficulties controlling my urge to engage in my studies,” and “我难以控制自己进行学业活动的冲动” in Chinese). Responses were rated on a 7-point Likert scale ranging from 1 (not agree at all) to 7 (very strongly agree). The harmonious passion and obsessive passion subscale scores were obtained by summing the respective items. Higher scores from two subscales indicated greater academic passions correspondingly. The Chinese version of the passion scale has demonstrated good reliability and validity (Zhao et al., 2015). In this study, Cronbach’s alphas were 0.87 for the harmonious passion, and 0.76 for the obsessive passion. Confirmatory factor analysis (CFA) also yielded satisfactory results: *χ^2^*(33) = 92.27, comparative fit index (CFI) = 0.98, root mean square error of approximation (RMSEA) = 0.05 (90% confidence interval between 0.04 and 0.06), and standardized root mean square residual (SRMR) = 0.03. More details about its reliability and validity are presented in Table 2.

**Table 2:** Reliability and validity of the passion scale.

Dimensions	Cronbach’s alpha	Composite reliability	Macdonald’s omega	Average variance extracted	Items	Factor loading
Harmonious passion	0.87	0.87	0.58	0.53	1, 3, 5, 6, 8, 10	0.69, 0.70, 0.67, 0.72, 0.80, 0.76
Obsessive passion	0.76	0.79	0.64	0.41	2, 4, 7, 9, 11, 12	0.48, 0.72, 0.76, 0.68, 0.80, 0.23

#### 2.2.2 Critical thinking

Critical thinking was assessed using the Chinese version of the California critical thinking disposition inventory (CCTDI-CV) (Facione et al., 1994; Yu and Yu, 2020). It was a six-dimensional scale consisting of 28 items. The dimension of truth-seeking ability included three items (e.g., “when analyzing a problem, I seek only facts that support my point of view,” “分析问题时, 我只寻求支持我观点的事实” in Chinese). The dimension of analyzing ability included three items (e.g., “if you disagree with someone else’s opinion, you must give reasons,” “要反对别人的意见就要提出理由” in Chinese). The dimension of systematization ability included five items (e.g., “I am good at developing orderly plans to solve complex problems,” “我善于制定有序的计划去解决复杂难题” in Chinese). The dimension of inquisitiveness included five items (e.g., “I like to explore how things work,” “我喜欢探究事物是如何运作的” in Chinese). The dimension of self-confidence included seven items (e.g., “when making decisions, others expect me to establish applicable criteria,” and “做决定时,别人会期待我制定适用的准则” in Chinese). The dimension of cognitive maturity included five items (e.g., “the best way to solve a problem is to ask others for the answer,” “解决难题的最好方法是向别人问取答案” in Chinese). The items were rated on a 6-point Likert scale ranging from 1 (not agree at all) to 6 (very strongly agree). The total score was obtained by summing the scores of 28 items, with higher score indicating more critical thinking. The CCTDI-CV has demonstrated good reliability and validity (Yu and Yu, 2020). In this study, the composite reliabilities for total score was 0.95. CFA also yielded satisfactory results: *χ^2^*(293) = 593.62, CFI = 0.95, RMSEA = 0.04 (90% confidence interval between 0.03 and 0.04), and SRMR = 0.04. More details about its reliability and validity are presented in Table 3.

**Table 3:** Reliability and validity of the CCTDI-CV.

Dimensions	Cronbach’s alpha	Composite reliability	Macdonald’s omega	Average variance extracted	Items	Factor loading
Truth-seeking	0.64	0.65	0.51	0.38	26, 27, 28	0.69, 0.66, 0.50
Analyzing	0.67	0.70	0.63	0.44	23, 24, 25	0.58, 0.81, 0.58
Systematization	0.78	0.82	0.56	0.53	13, 14, 15, 16, 17	0.76, 0.81, 0.81, 0.86, 0.10
Inquisitiveness	0.82	0.82	0.60	0.48	8, 9, 10, 11, 12	0.69, 0.79, 0.62, 0.74, 0.62
Self-confidence	0.82	0.83	0.61	0.41	1, 2, 3, 4, 5, 6, 7	0.67, 0.69, 0.69, 0.64, 0.68, 0.57, 0.52
Cognitive maturity	0.68	0.68	0.58	0.30	18, 19, 20, 21, 22	0.49, 0.51, 0.51, 0.59, 0.61

### 2.3 Data analysis

Data analysis was conducted in several stages, which are detailed below:

For preliminary analysis, descriptive statistics and Pearson correlations were computed for all variables using SPSS. Internal consistency for all scales was assessed using Cronbach’s alpha, composite reliability (CR), and Macdonald’s Omega. These metrics ensure the reliability of the measures, with CR and Omega providing a more accurate estimate of consistency by considering factor loadings and measurement error, which were extracted directly from Mplus. The fit indices used were comparative fit index (CFI > 0.90), root mean square error of approximation (RMSEA <0.10), and standardized root mean square residual (SRMR <0.08) (Marsh et al., 2005). Convergent validity was assessed using average variance extracted (AVE), with a threshold of AVE > = 0.50 indicating sufficient shared variance among items of the same construct.

Then, LPA was performed in Mplus to identify latent profiles of harmonious and obsessive passions. To determine the optimal profiles for harmonious and obsessive passions, we used the log likelihood (LL), Akaike information criterion (AIC), Bayesian information criterion (BIC), sample size adjusted BIC (SSA-BIC), entropy, adjusted Lo-Mendel-Rubin likelihood ratio test (aLMR), and bootstrapped likelihood ratio test (BLRT) (Ferguson et al., 2020; Nylund-Gibson and Choi, 2018). For the information-based criteria, lower values for the k-profiles solution indicate that the k-profiles solution outperforms the k-1-profiles solution. An entropy value greater than 0.80 indicates “good” classification of individual cases into classes (Nylund-Gibson and Choi, 2018). For aLMR and BLRT, a non-significant *p* value (*p* > =0.05) for the k-profiles solution implies that the k-profiles solution is not superior to the k-1-profiles solution.

After determining the optimal latent profiles, the BCH procedure (Bakk and Vermunt, 2016) in Mplus was applied to test differences in critical thinking among the profiles. The BCH method accounts for classification uncertainty, providing robust comparisons between profiles.

All analyses were conducted in SPSS version 25.0 (descriptive statistics, reliability) and Mplus version 8.6 (Muthén and Muthén, 2008–2021) (CFA, factor loadings, LPA, BCH). Mplus was chosen for its robust handling of latent variable models and profile analyses, ensuring precise and reliable results.

## 3 Results

### 3.1 Preliminary analysis

Descriptive statistics and correlations among academic passions and critical thinking are presented in Table 4. Harmonious passion correlated moderately and significantly with obsessive passion and critical thinking. However, obsessive passion exhibited a weak yet significant correlation with critical thinking.

**Table 4:** Descriptive statistics and correlations among academic passions and critical thinking.

	*M*	*SD*	1	2	3	4	5	6	7	8	9
1.Harmonious passion	26.42	6.10	1								
2.Obsessive passion	16.89	5.52	0.41***	1							
3.Critical thinking	112.80	13.63	0.56***	0.12**	1						
4.Truth-seeking	11.60	2.57	−0.03	−0.37***	0.29***	1					
5.Analyzing	13.21	2.41	0.35***	0.13**	0.42***	−0.17***	1				
6.Systematization	19.51	4.13	0.50***	0.21***	0.78***	0.04	0.30***	1			
7.Inquisitiveness	20.49	4.25	0.54***	0.30***	0.74***	−0.06	0.34***	0.51***	1		
8.Self-confidence	27.14	5.17	0.51***	0.30***	0.76***	−0.09*	0.32***	0.56***	0.61***	1	
9.Cognitive Maturity	20.85	4.07	−0.03	−0.36***	0.39***	0.55***	−0.12**	0.13***	−0.02	−0.07	1

*p < 0.05, **p < 0.01, ***p < 0.001.

M, mean; SD, standard deviation.

### 3.2 Latent profiles for harmonious and obsessive passions

**Figure 1:**
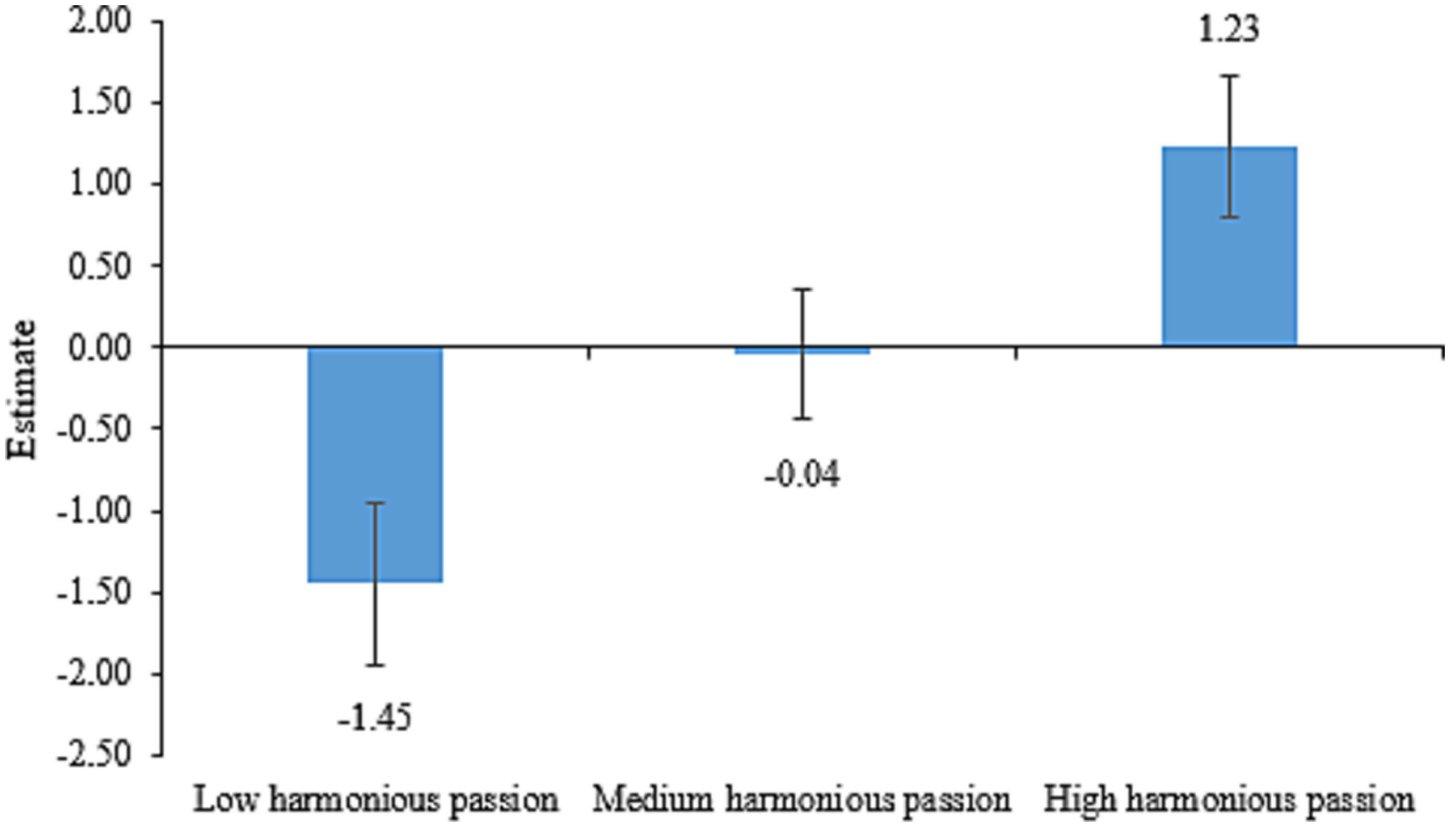
Latent profiles of harmonious passion.

The LPA was performed using items from harmonious and obsessive passions as input. Table 5 presents fit statistics for harmonious passion. As profiles decreased, AIC, BIC, and SSA-BIC all reduced. The entropy index favored the three-profile solution (>0.80). Additionally, aLMR did not reach significance for the four-profile solution, indicating superiority of the three-profile solution as well. Thus, we adopted the three-profile solution. The estimates for three latent profiles of harmonious passion are shown in Figure 1. The first latent profile, which consisted of 142 respondents (20.34%), was characterized by the smallest estimate. We called this profile “low harmonious passion.” The second latent profile, which consisted of 376 respondents (53.87%), was characterized by the moderate estimate. We called this profile “medium harmonious passion.” And the third latent profile, which consisted of 180 respondents (25.79%), was characterized by the greatest estimate. We called this profile “high harmonious passion.”

**Table 5:** Fit statistics from the latent profile analysis for harmonious passion.

*N* of profiles	LL	FP	AIC	BIC	SSA-BIC	Entropy	aLMR (*p*)	BLRT (*p*)
1	−7064.12	12	14206.82	14168.72				
2	−6485.80	19	13009.60	13096.02	13035.69	0.79	0.00	0.00
3	−6265.93	26	12583.86	12702.11	12619.56	0.82	0.00	0.00
4	−6221.29	33	12508.57	12658.66	12553.88	0.78	0.19	0.00
5	−6193.84	40	12467.68	12649.61	12522.60	0.80	0.05	0.00

LL, log likelihood; FP, free parameters; AIC, Akaike information criterion; BIC, Bayesian information criterion; SSA-BIC, sample size adjusted BIC; aLMR, adjusted Lo–Mendel–Rubin likelihood ratio test; BLRT, bootstrapped likelihood ratio test.

**Figure 2:**
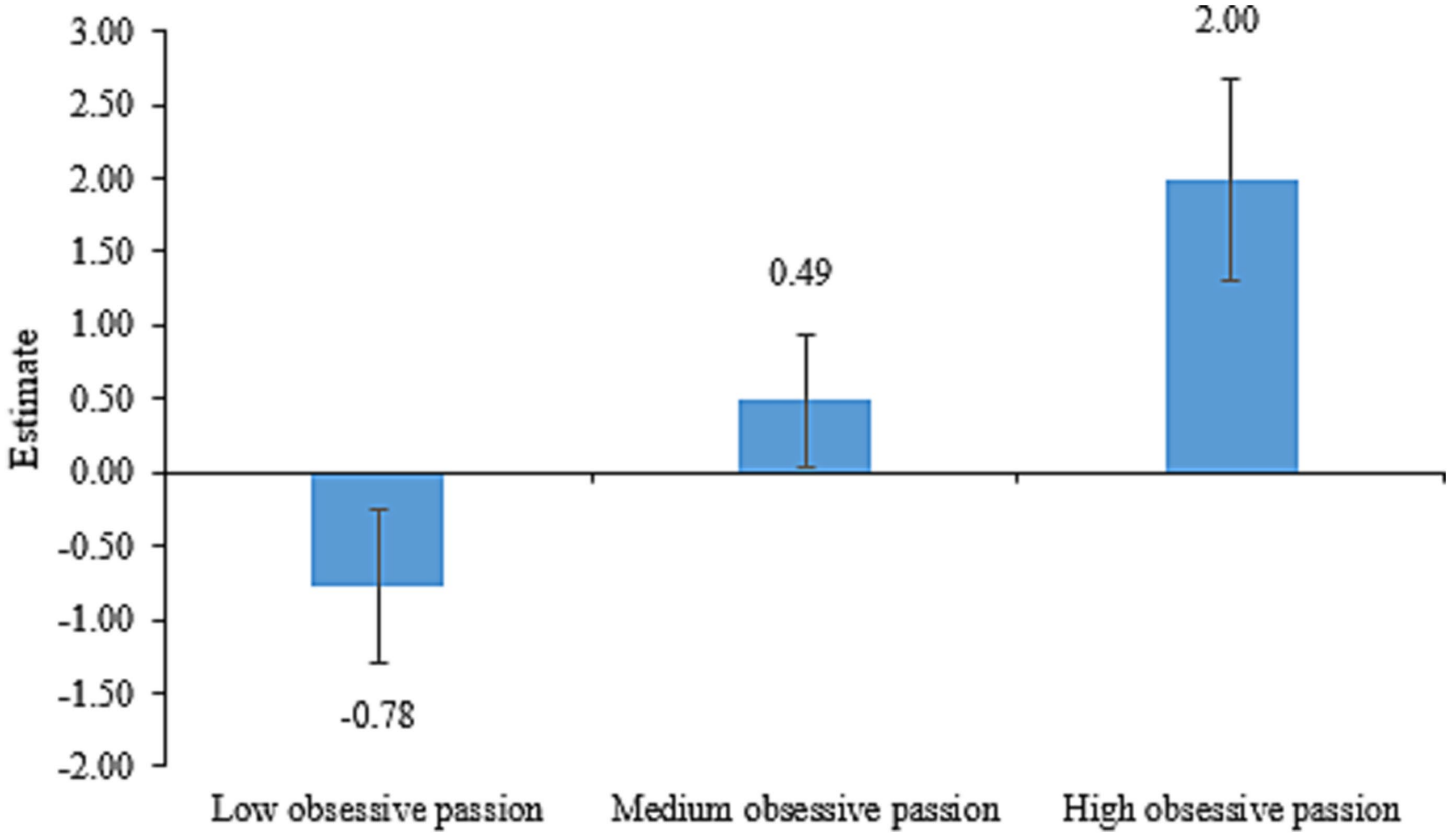
Latent profiles of obsessive passion.

Similarly, Table 6 presents the fit statistics for obsessive passion. AIC, BIC, and SSA-BIC decreased with fewer profiles. The entropy index favored the three- and five-profile solutions (>0.80). Additionally, aLMR was not significant for the four- and five-profile solutions, indicating that the four-profile solution did not outperform the three-profile solution and the five-profile solution did not outperform the four-profile solution. Therefore, we adopted the three-profile solution. The estimates for three latent profiles of obsessive passion are shown in Figure 2. The first latent profile, which consisted of 340 respondents (48.71%), was characterized by the smallest estimate. We called this profile “low obsessive passion.” The second latent profile, which consisted of 298 respondents (42.69%), was characterized by the moderate estimate. We called this profile “medium obsessive passion.” And the third latent profile, which consisted of 60 respondents (8.60%), was characterized by the greatest estimate. We called this profile “high obsessive passion.”

**Table 6:** Fit statistics from the latent profile analysis for obsessive passion.

*N* of profiles	LL	FP	AIC	BIC	SSA-BIC	Entropy	aLMR (*p*)	BLRT (*p*)
1	−7187.06	12	14398.12	14452.70	14414.60	–	–	–
2	−6735.41	19	13508.82	13595.23	13534.90	0.80	0.00	0.00
3	−6595.77	26	13243.54	13361.79	13279.23	0.83	0.00	0.00
4	−6544.82	33	13155.64	13305.73	13200.95	0.80	0.09	0.00
5	−6495.96	40	13071.91	13253.84	13126.83	0.82	0.08	0.00

LL, log likelihood; FP, free parameters; AIC, Akaike information criterion; BIC, Bayesian information criterion; SSA-BIC, sample size adjusted BIC; aLMR, adjusted Lo–Mendel–Rubin likelihood ratio test; BLRT, bootstrapped likelihood ratio test.

### 3.3 Outcomes of latent profile membership

The relationships between two academic passions and critical thinking are illustrated in Tables 7, 8, respectively. The BCH procedure examined whether there were significant differences in critical thinking across three latent profiles. The results indicated that the respondents in the “high harmonious passion” profile demonstrated the highest critical thinking ability, whereas those in the “low harmonious passion” profile exhibited the lowest. The differences of critical thinking across three latent profiles were significant. On the other hand, the differences of critical thinking across three latent profiles of obsessive passion displayed similar pattern. The respondents in the “high obsessive passion” profile showed the highest critical thinking ability, contrasting with the lowest in the “low obsessive passion” profile. Notably, the differences of critical thinking across three latent profiles were significant. To be mentioned, the differences in critical thinking were more pronounced among harmonious passion profiles compared to obsessive passion profiles.

**Table 7:** Equality tests of means across harmonious passion profiles for critical thinking (BCH).

	1. Low harmonious passion	2. Medium harmonious passion	3. High harmonious passion	Chi-square	Significant differences
Critical thinking	101.92	111.52	123.60	204.44	1 < 2 < 3

All values of critical thinking are raw scores.

**Table 8:** Equality tests of means across obsessive passion profiles for critical thinking (BCH).

	1. Low obsessive passion	2. Medium obsessive passion	3. High obsessive passion	Chi-square	Significant differences
Critical thinking	110.91	113.87	117.91	15.91	1 < 2 < 3

All values of critical thinking are raw scores.

## 4 Discussion

To the best of our knowledge, this study is the first to investigate the relationship between academic passions and critical thinking using LPA. And we uncovered several meaningful and interesting results, which are discussed as follow.

### 4.1 Harmonious academic passion and obsessive academic passion

Traditionally, high harmonious passion was thought to preclude engagement in activities driven by obsessive passion (Rahimi et al., 2023). However, our study revealed that harmonious and obsessive academic passions were significantly positively correlated with each other, consistent with prior research (Peixoto et al., 2021; Pollack et al., 2020; Sverdlik et al., 2022). In other words, this suggests that harmonious passion and obsessive passion are not dualistic opposites strictly.

Several explanations are proposed. First, both harmonious passion and obsessive passion stem from strong interest and commitment to an activity, though the former emphasizes the controllable, positive aspects while the latter emphasizes the uncontrollable, negative aspects (Paquette et al., 2023; Rahimi et al., 2023; Vallerand et al., 2003). Secondly, harmonious passion and obsessive passion may share early development origins rooted in high levels of interest and dictation to the activity. They are all linked to an individual’s psychological traits and behavioral patterns (Sverdlik et al., 2022; Vallerand and Paquette, 2024). Lastly, social and cultural contexts may also contribute to the shift from harmonious passion to obsessive passion. Particularly, in a competitive environment, individuals may feel external pressures that may push harmonious passion toward obsessive passion. Both harmonious and obsessive passions were found to positively correlate with critical thinking (Peixoto et al., 2021; Sverdlik et al., 2022), supporting the abovementioned explanations and the presence of three latent profiles for each passion type.

Despite these similarities mentioned above, the proportions of the three latent profiles for these harmonious and obsessive academic passions differed notably (20.34, 53.87, and 25.79% for harmonious passion versus 48.71, 42.69, and 8.60% for obsessive passion). Hypothesis 1 has been proved in this study. This suggests that while harmonious and obsessive passions were positively correlated, they may represent distinct dimensions, aligning with the dualistic model of passion theory (Paquette et al., 2023; Rahimi et al., 2023). Additionally, harmonious passion better discriminates respondents (Amarnani et al., 2020) based on varying levels of critical thinking, as seen in Tables 5, 6 where the mean differences of critical thinking across harmonious passion profiles exceeded those across obsessive passion profiles.

### 4.2 Relationship between academic passions and critical thinking

This study explored the relationship between two types of academic passion (harmonious passion and obsessive passion) and critical thinking ability. The results showed that students in the “high harmonious passion” profile have the highest critical thinking ability, while students in the “low harmonious passion” profile have the lowest critical thinking ability. The obsessive passion profiles showed a similar trend: critical thinking skills were highest in the “high obsessive passion” profile and lowest in the “low obsessive passion” profile. In addition, the harmonious passion profiles showed more significant differences in critical thinking than the obsessive passion profiles. Hypothesis 2 has been proved in this study. This finding is consistent with existing research, and many scholars believe that passion, especially harmonious passion, can promote students’ cognitive engagement and emotional investment (Lavoie et al., 2021; Paquette et al., 2023; St-Louis et al., 2021), and then it improves critical thinking abilities. Harmonious passion enables students to maintain a high degree of motivation and interest in academic exploration, thereby stimulating deeper thinking and logical reasoning (Gkorezis et al., 2021; Zhang et al., 2022). Therefore, students with higher harmonious passion show stronger critical thinking skills when faced with academic problems (Chang et al., 2022; Namaziandost et al., 2023).

Obsessive passion is not always harmful to critical thinking. The correlation between obsessive passion and critical thinking was significantly positive, though low (Peixoto et al., 2021; Pollack et al., 2020; Sverdlik et al., 2022). And it could distinguish respondents across three latent profiles of critical thinking, just as harmonious passion did. This underscores new avenue for researchers and practitioners alike. Nevertheless, the effects of obsessive academic passion on critical thinking ability are more complex. Although students from high obsessive academic passion profile also showed strong critical thinking ability, obsessive academic passion is often associated with psychological stress and over-commitment (Cheyroux et al., 2025), which may lead to burnout or anxiety in learning (Lobato et al., 2023; Rahimi and Vallerand, 2021), as a result, affecting their long-term academic performance. Therefore, how to balance harmonious and obsessive academic passions to prevent students from being overly addicted to a certain field, has become a question that needs to be further explored in future research and educational practice.

Overall, this study is consistent with previous research showing that passion, especially academic passion, plays a key role in the development of students’ critical thinking abilities. Two main limitations of the study design require clarification. Firstly, being a cross-sectional study, we cannot establish that among the academic passions and critical thinking, which is the independent variable and which is the dependent variable. Future research should use longitudinal data or conduct experiments to establish causal relationship among harmonious passion, obsessive passion and critical thinking, and to elucidate their mechanisms of formulation and change. Secondly, the study’s conclusions are based on a sample of college students, most of whom are female, highlighting the need for validation with other demographics in future, such as males, middle school students and middle-aged adults, to ensure broader generalizability. Moreover, future research can further explore the relationships and differences in academic passions and critical thinking between Eastern and Western cultures.

## 5 Conclusion

This study confirms the heterogeneity of harmonious and obsessive passions among college students. Three latent profiles for harmonious and obsessive passions are obtained, which are “low harmonious passion,” “medium harmonious passion,” and “high harmonious passion” for harmonious passion, “low obsessive passion,” “medium obsessive passion,” and “high obsessive passion” for obsessive passion, respectively. Students with higher levels of academic passions demonstrate enhanced critical thinking abilities compared to their peers.

According to abovementioned results, this study offers several implications for practice. First, colleges and universities should provide targeted counseling and guidance based on the types of students’ passion. For students with low harmonious passion, carefully designed tasks that balance guidance and autonomy can help them gradually experience intrinsic satisfaction from learning. Second, although obsessive passion may drive students’ short-term efforts, it may lead to excessive stress or burnout in the end. Therefore, it is necessary to balance the relationship between harmonious and obsessive passions to promote students’ mental health and academic persistence. Third, colleges and universities should design courses and activities that encourage personal interest and engagement, helping students develop harmonious passion and improve their critical thinking ability. In short, this study emphasizes the importance of understanding and guiding academic passion in education management and provides a practical basis for improving students’ comprehensive abilities.

## Data Availability

The raw data supporting the conclusions of this article will be made available by the authors, without undue reservation.
